# Informing robot design through early public engagement: lay perceptions of soft versus rigid socially assistive and rescue robots

**DOI:** 10.3389/frobt.2026.1741946

**Published:** 2026-03-19

**Authors:** J. Fenn, L. Estadieu, M. Gorki, I. Monno, F. Tauber, J. Teichmann, S. Levy-Tzedek, T. Speck, O. Müller, A. Kiesel

**Affiliations:** 1 Cluster of Excellence livMatS @ FIT Freiburg Center for Interactive Materials and Bioinspired Technologies, University of Freiburg, Freiburg, Germany; 2 Institute of Psychology, University of Freiburg, Freiburg, Germany; 3 Department for Humanities, Social and Political Sciences, ETH Zürich, Zürich, Switzerland; 4 Recanati School for Community Health Professions, Department of Physical Therapy, Faculty of Health Sciences, Ben-Gurion University of the Negev, Beer-Sheva, Israel; 5 Freiburg Institute for Advanced Studies (FRIAS), University of Freiburg, Freiburg, Germany; 6 Plant Biomechanics Group @ Botanic Garden Freiburg, University of Freiburg, Freiburg, Germany; 7 Faculty of Philosophy, University of Freiburg, Freiburg, Germany

**Keywords:** design evaluation, multi-method study, perceived risks and benefits, public perception, search and rescue robots, socially assistive robots, soft robots

## Abstract

As soft robots become more prevalent in society, it becomes increasingly important to understand how laypersons evaluate their risks and benefits relative to conventional rigid robots. This article investigates public perceptions of soft versus rigid embodiments of socially assistive robots (SAR) and rescue robots (RR) and explores how these perceptions can inform early-stage robot design. We conducted an online study, using a scenario-based intervention design combined with Cognitive-Affective Maps (CAMs) to capture participants’ cognitive–emotional belief structures. In a first step, participants constructed CAMs depicting perceived risks and benefits of rigid SAR or RR. After reading a second scenario introducing the corresponding soft robot, they revised their maps, allowing a direct contrastive comparison between the first (rigid) and second (soft) scenario. Quantitative analyses showed that, across both application domains, post-intervention evaluations (after the soft-robot scenarios) were more positive than pre-intervention evaluations of rigid robots. Qualitative analyses revealed distinct argument structures: After learning about soft robots, participants added concepts emphasizing safety, emotional comfort, and adaptability, but also introduced concerns such as fragility and emotional dependence, whereas rigid robots were linked to precision, robustness, and efficiency, alongside worries about technical failure, data security, and emotional detachment. By integrating intervention-based CAMs with data-driven qualitative synthesis, the study demonstrates a scalable method for early public engagement that uncovers how laypersons qualitatively negotiate trade-offs between soft and rigid designs in plausible early-stage scenarios. These insights provide actionable input for human-centered design of soft robots, supporting responsible and socially aligned robot development.

## 1 Introduction

Soft robotics promises safer and more adaptive human–robot interaction due to the intrinsic mechanical compliance and flexibility of soft materials, which reduce the risk of injury, enable physical contact, and allow robust operation in unstructured or confined environments (Lee et al., 2017; Yasa et al., 2023). Soft and rigid robotics differ fundamentally in material composition, mechanical behavior, and interaction principles: Rigid robots, typically built from high-stiffness metals or composites, provide high precision, load capacity, and mature control architectures, making them ideal for structured industrial settings (Alici, 2018; Yasa et al., 2023). In contrast, soft robots are constructed from compliant materials inspired by biological systems, enabling continuous deformation, morphological adaptability, and embodied intelligence in complex and dynamic environments (Kim et al., 2013; Tauber et al., 2023; Trivedi et al., 2008; Wang and Chortos, 2022). Beyond their mechanical compliance, additional characteristics such as improved energy efficiency, lower computational demands for gripping tasks, reduced production costs, and potential biodegradability further enhance their suitability for applications requiring physical safety, environmental adaptability, and human-centered interaction (Milana, 2022; Rossiter, 2021; van Adrichem and Jovanova, 2021). First systems such as *RoBoa* for search-and-rescue operations (der Maur et al., 2021) and the *HugBot* for socially assistive care (Hedayati et al., 2019) exemplify how compliant design principles can extend robotic functionality to delicate or unpredictable settings. With ongoing advances in materials, actuation, and control, the potential of soft robotics is expected to expand substantially (Liu et al., 2023; Mazzolai et al., 2022; Tauber et al., 2023; Trivedi et al., 2008; Yasa et al., 2023).

As the field of soft robotics advances toward real-world deployment, integrating user perception data into early design stages is essential to ensure that the advantages of compliance, safety, and adaptability translate into socially accepted and functionally effective systems. Existing research has addressed individual perceptual dimensions of soft robotics, such as perceived naturalness (Jørgensen et al., 2022), emotional valence during interaction (Jørgensen, 2023), stress and anxiety reduction through visual or tactile exposure (Probst et al., 2024), and safety perception influenced by motion dynamics (Wang et al., 2024). Complementary studies grounded in technology acceptance frameworks, including the Almere Model (Heerink et al., 2010) and the Technology Acceptance Model (Venkatesh and Bala, 2008), have identified perceived usefulness, ease of use, enjoyment, trust, and self-efficacy as major determinants of robot acceptance (Felding et al., 2023; Whelan et al., 2018; Latikka et al., 2019). Generally, people tend to hold positive attitudes toward social robots (David et al., 2022), although acceptance is further shaped by user characteristics such as loneliness or familiarity with robots (Bishop et al., 2019). An expert survey pointed out barriers including technical limitations (e.g., uncontrollability), lack of emotional expressiveness, and security concerns (Moradi et al., 2018). Moreover, societal analyses highlight concerns about automation-related job insecurity, increased technological dependence, and excessive skill demands (Yam et al., 2023; Meissner et al., 2020).

However, these studies typically capture static evaluations or isolated constructs and rarely examine how laypersons qualitatively conceptualize and emotionally negotiate the trade-offs between soft and rigid robot designs. Our study addresses this gap by introducing an intervention-based Cognitive-Affective Mapping approach that systematically reveals the conceptual argument structures and emotional dynamics towards rigid and soft robot designs (see Section 1.1).

### 1.1 Innovative study design to identify central argument structures of soft robots

**FIGURE 1:**
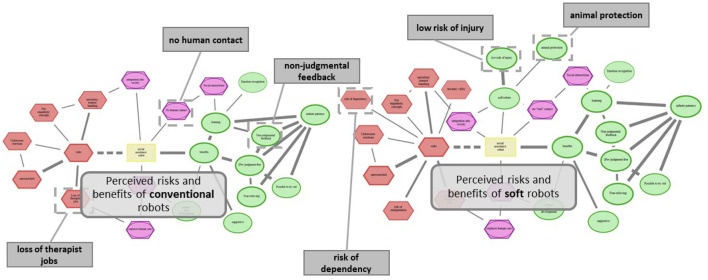
Graphical representation of layperson perceptions of (soft) socially assistive robots. By addressing laypersons’ attitudes toward soft robots, the study aims to promote responsible innovation and informed design decisions applicable to both socially assistive robots (SAR) and rescue robots (RR).

Our study introduces an innovative approach to early-stage design evaluation by combining intervention-based experimentation with Cognitive-Affective Maps (CAMs), a mind-map like method (Fenn et al., 2025). This mixed-method framework enables the systematic visualization and quantification of how participants cognitively and emotionally represent perceived risks and benefits of robotic systems, as shown in prior discussions of the methodology’s usefulness (Livanec et al., 2022; Möller et al., 2021; Reuter et al., 2022) and its application to emerging climate engineering technologies (Fenn et al., 2023). Unlike conventional survey-based approaches that assess predefined or static attitudes, CAMs uncover the underlying argument structures, emotional valences, and conceptual shifts that emerge when laypersons are introduced to new robotic embodiments. By applying this method to both socially assistive robots (SAR) and rescue robots (RR), we address two domains in which soft robotics holds particularly high societal relevance. Soft SARs have the potential to enhance emotional wellbeing, physical support, and rehabilitation outcomes through safe and adaptive physical interaction, making them valuable in healthcare, eldercare, and therapeutic contexts (Boada et al., 2021; Langer et al., 2019; Langer and Levy-Tzedek, 2021). In contrast, soft RRs can improve operational safety and efficiency in hazardous or unpredictable environments by navigating debris, adapting to uneven terrain, and performing delicate tasks such as locating or assisting trapped individuals (Battistuzzi et al., 2021; Chitikena et al., 2023; Zuzánek et al., 2014). The study provides a scalable framework for integrating user perceptions into the formative stages of soft robot design, promoting responsible innovation and early societal alignment (Kaplan et al., 2021; Stilgoe et al., 2013). Figure 1 illustrates how layperson evaluations can inform design considerations for socially interactive and safety-critical robotic systems.

**FIGURE 2:**
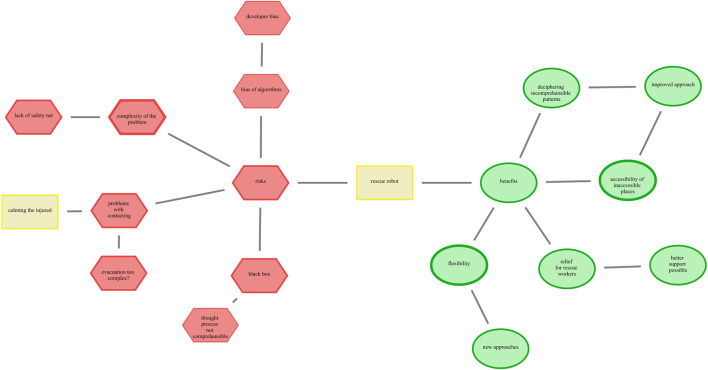
Exemplary CAM on perceived risks and benefits of soft RR drawn by one participant. Valences of the concepts are represented by the concepts’ shapes and colors. Green ovals = positive affect; red hexagons = negative affect; yellow rectangles = neutral affect; purple superimposed hexagons with ovals = ambivalent affect. For green and red colored concepts, strength of the shape’s border denotes a grading of the emotional evaluation ranging from [-3,3] – the thicker the frame, the more positive/negative the concept. Two types of links indicate the relations between concepts. Solid lines = supportive connections; dashed lines = inhibiting connections.

To evaluate how people perceive soft robots compared to rigid robots, we conducted an online study with a final sample of 180 participants. The study was designed to assess perceived risks and benefits using a scenario-based intervention framework grounded in method of Cognitive-Affective Mapping (Thagard, 2010). CAMs are a structured, mind-map-like methodology for representing and analyzing participants’ cognitive and emotional associations toward a given topic (Homer-Dixon et al., 2013; Reuter et al., 2022). Extending traditional mind-mapping techniques, CAMs enable both qualitative and quantitative analyses of belief structures by integrating cognitive relations and affective evaluations (Fenn et al., 2025). Concepts are interconnected through weighted supportive or inhibitory links and assigned emotional valence ratings on a scale from −3 (very negative) to +3 (very positive), which are visualized using color coding (green = positive, red = negative, yellow = neutral, purple = ambivalent). Using our custom-developed software (Fenn et al., 2025), participants constructed CAMs centered on predefined core concepts—either “socially assistive robot” (SAR) or “rescue robot” (RR)—with “benefits” positioned on the right and “risks” on the left. Participants were instructed to draw additional concepts, link them, assign emotional evaluations, and provide short explanatory comments where appropriate. An exemplary CAM drawn by a participant, illustrating the perceived risks and benefits of a soft RR, is shown in Figure 2.

**FIGURE 3:**
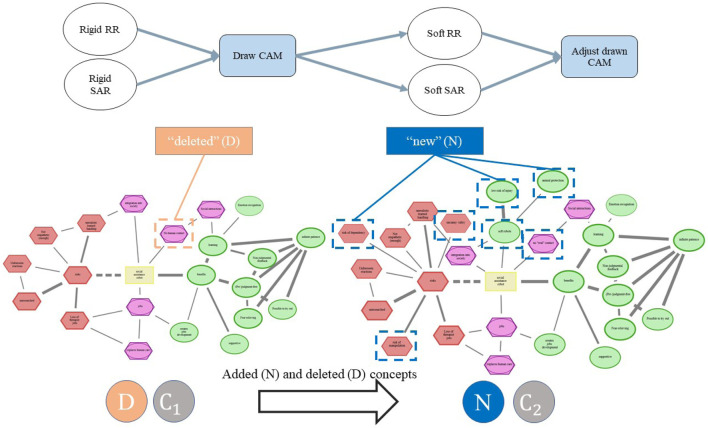
Evolution of CAMs in study design depicts the construction and updating of a CAM to reflect their perceptions of risks and benefits. This example shows a CAM for a SAR with added (“soft”) and removed (“rigid”) concepts in the post-intervention.

The intervention design involved a two-step mapping process. In the first phase (pre-intervention), participants read a scenario text describing either a conventional rigid SAR or RR and created an initial CAM representing the perceived risks and benefits of that robot type. In the second phase (post-intervention), participants were exposed to a follow-up scenario describing the corresponding soft robotic version and were asked to revise their CAMs accordingly. This included modifying existing concepts, adjusting their emotional valence, or adding and removing concepts to reflect newly perceived risks and benefits. The resulting CAMs were then analyzed by categorizing each concept as *constant* (unchanged), *deleted*, or *new* (added after the intervention). Figure 3 illustrates this process with an exemplary CAM, showing newly added concepts such as “low risk of injury” (positive) and removed elements like “no human contact” (negative), reflecting how exposure to information about soft robotics can reshape lay perceptions. For readability, in the results, constant concepts (pre-intervention) are labeled as “rigid”, while new concepts (post-intervention) are labeled as “soft”.

However, this labeling reflects temporal order rather than robot-specific associations. A concept labeled “rigid” may equally apply to soft robots but was simply mentioned before the intervention. Consequently, all interpretations of “rigid” versus “soft” concepts should be understood as reflecting pre-versus post-intervention mentions, rather than mutually exclusive robot-type attributions. Because the study employs sequential exposure (rigid first, soft second) and a comparative framing (explicit instruction to revise CAMs), observed differences are interpreted in the following as contrastive evaluations shaped by the task context, rather than independent assessments of each embodiment in isolation (Rosenthal-von der Pütten et al., 2013; Torre and White, 2021). In general, such within-participants designs are prone to order effects (e.g., familiarity, fatigue, learning/habituation, or novelty effect), which can influence the evaluations independent of the manipulated content, whereby counterbalancing is a standard remedy, whereas fixed-order designs (as used here) require cautious interpretation (Hoffman and Zhao, 2020; Rosenthal-von der Pütten et al., 2025). Hereby, consistent with qualitative, design-oriented HRI research our study is explicitly design-oriented (Veling and McGinn, 2021): we use revision-based CAMs to elicit the considerations, trade-offs, and central attributes that laypersons foreground when comparing alternative robot embodiments in plausible early-stage scenarios.

By automatically classifying the concepts in participants’ CAMs as constant, deleted, or newly generated, we quantify revision-based shifts in which risks, benefits, and interaction-relevant themes participants emphasize after sequential exposure to a rigid-robot scenario followed by a soft counterpart scenario. These shifts are interpreted as contrastive, scenario-dependent evaluations, and serve as actionable input for early design recommendations (see Table 1). Our study was guided by three key research questions, which have been investigated in the respective result sections (see Section 3):

1. Are soft robots perceived more positively than rigid robots?
2. What are the most significant risks and benefits associated with soft robots compared to conventional robots?
3. To what extent do demographic factors, such as gender, shape the evaluation and perception of soft robots compared to rigid robots?

**TABLE 1:** Comparison of participant-derive design considerations under scenario-based, contrastive elicitation for RR and SAR regarding perceived risks and safety, and human-robot interaction dynamics as input for early-stage design recommendations.

Category	Facet	Rescue robots	Socially assistive robots
Perceived risks, safety	Differences (soft vs. rigid)	**Soft robots:** Require durable, compliant materials to address fragility and reduce risks of damage, minimizing injury risks through flexibility in interaction **Rigid robots:** Emphasize strength and redundancy to counter risks from technical failure, developmental errors, and ethical misuse	**Soft robots:** Leverage flexible materials to reduce injury risks but require enhanced fail–safe systems to address processing vulnerabilities **Rigid robots:** Address concerns about data security and potential physical harm from rigid structures
	Most important	Prioritize safety by ensuring reliable, predictable, and user–centered operation across all robot types	Combine soft materials with robust fail–safes and predictable behavior to enhance safety and reliability in socially assistive robots
Perceived negative, positive human robot interaction	Differences (soft vs. rigid)	**Soft robots:** Better suited for fostering emotional connections due to flexible, approachable materials and designs that minimize fear and discomfort. Enable safer physical interactions and adaptability in complex environments but must address precision concerns to ensure reliability **Rigid robots:** Prioritize durability, operational stability, and physical robustness to withstand harsh conditions and extended missions. Their mechanical appearance may evoke emotional detachment	**Soft robots:** Emphasize comfort through soft, tactile–friendly materials to enhance emotional engagement and minimize physical intimidation, but must address concerns of over–dependence, fragility, and maintenance challenges **Rigid robots:** Prioritize robustness and technical precision to counter perceptions of fear or intimidation while ensuring safe operation in structured environments
	Most important	Incorporate empathetic interaction and safety mechanisms to ensure reliability and emotional support during rescue operations	Balance emotional understanding and physical safety while ensuring reliable, user–centered operation across both robot types

## 2 Methods

### 2.1 Procedure

**FIGURE 4:**
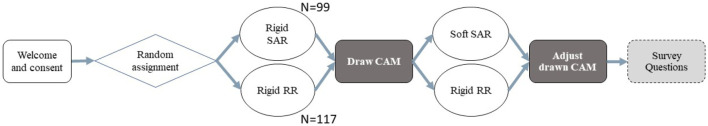
Study flow diagram illustrates the six-step study process, where ellipses represent the scenario texts and the drawn CAMs for both rigid and soft robots are colored in dark gray. The survey questions, shown in light gray, were analyzed in an exploratory manner.

We employed a mixed-method approach to investigate the perceptions of SAR and RR through a structured six-step process, as illustrated in Figure 4. (1) Initially, participants were welcomed and provided consent to partake in the study. (2) To ensure thoughtful participation, a seriousness check was implemented before participants were randomly assigned to receive a scenario text outlining the potential risks and benefits of either conventional SAR or RR. (3) Subsequently, participants were introduced to the CAM software and instructed to construct a CAM depicting their perceived risks and benefits of the assigned robot type. (4) After this initial drawing phase, they were presented with a second scenario text describing the soft counterpart of their initially assigned robot—either a soft SAR or soft RR. (5) Participants then revised their CAM based on the newly acquired information, thereby eliciting a contrastive evaluation in explicit comparison to the previously presented rigid-robot scenario. (6) Finally, they completed a set of survey questions designed to assess their overall perception of robots (6). The study flow diagram in Figure 4 visually represents this process, with ellipses indicating the scenario texts, dark gray boxes denoting the drawn CAMs for both rigid and soft robots, and light gray indicating the survey questions, whereby only the CAM data is reported in this article.

#### 2.1.1 Development of scenario texts

These scenario texts were developed by two postdoctoral researchers with expertise in psychology and ethics, following a review of the robotics literature to identify key risks and benefits associated with conventional and soft SAR and RR. This process was iterative, creating different versions of the scenario texts in accordance with recommendations from existing research (Kosow and Gassner, 2008; Schwartz, 1996). Quality criteria such as readability, plausibility, and consistency were considered to ensure the scenarios were accurately depicted (Kosow and Gassner, 2008; Mietzner and Reger, 2005). Additionally, the scenario texts were informed by an expert survey involving 21 specialists in soft robotics, materials science, and related fields, all affiliated with the Cluster of Excellence Living Materials Systems (*liv*MatS) at the University of Freiburg (Speck et al., 2023). These experts provided insights into the primary risks and benefits of both conventional and soft SAR and RR (see in detail “1. Feedback Experts” in the Supplementary Material). The final scenario texts described either the conventional rigid SAR or RR and their soft counterparts. Each scenario provided general information, followed by three potential risks and three potential benefits, ensuring a balanced presentation of information.

### 2.2 Collected Cognitive-Affective Maps

As described in Sections 1.1, 2.1, participants first constructed a CAM based on either a SAR or a RR. Each CAM was centered around a predefined core concept—either “socially assistive robot” or “rescue robot”—with “benefits” positioned to the right and “risks” to the left. Using our custom-developed CAM software (Fenn et al., 2025), participants were instructed to add at least ten new concepts, connect them through supportive or inhibitory links, and provide brief explanatory comments. The predefined core concepts remained fixed to ensure comparability across participants. After completing their initial CAM, participants rated its representativeness on a seven-point Likert scale (1 = completely unrepresentative, 7 = fully representative), yielding an average score of 6.02 (SD = 0.93), indicating that participants perceived their initial drawn CAMs as highly reflective of their own attitudes and thoughts. Subsequently, participants read a second scenario describing the soft counterpart of their initially assigned robot (either a soft SAR or soft RR) and were instructed to revise their CAMs by adding, deleting, or modifying concepts to reflect their contrastive evaluations. The resulting changes were automatically categorized as “new”, “deleted”, or “constant”.

Data were collected from 226 participants in Germany via the online platform Prolific in December 2023. In total, 452 CAMs were drawn, comprising one map for rigid and one for soft robots per participant. We excluded eight persons (16 CAMs) from further analysis for several reasons: One participant created too many empty concepts, another only used predefined concepts, and a third predominantly added the concept with the written text “empty”. Additionally, five participants encountered technical issues, likely due to slow internet connections, which prevented the default CAM with predefined concepts from loading correctly. After these exclusions, our sample consisted of 436 CAMs (respective 218 participants), resulting in a data loss of approximately 3.5% due to human and technical errors. While the majority of participants (83%) introduced new concepts, approximately 17% neither added nor removed any concepts, despite explicit instructions. These participants were excluded from further analysis, because our planned analyses quantify revision-related changes. However, we acknowledge that “no change” may represent a meaningful outcome (e.g., stability or resistance to updating) rather than noncompliance, and excluding such cases may upwardly bias observed change patterns. This resulted in a final sample of 180 participants, each of whom created an initial CAM reflecting their perceptions of rigid robots and later revised it to incorporate their updated perceptions of soft robots, yielding 98 participants for RR and 82 for SAR. In the final dataset of 360 CAMs, whereby ambivalent and neutral concepts were assigned a value of 0, the mean valence was slightly positive with 0.19 (SD = 0.47). Participants drew an average of 15.06 concepts per CAM (SD = 3.72), resulting in 1,902 unique concepts (5,420 total) and 1,113 comments, with an average length of 11.85 words (SD = 7.33). The most frequently drawn concepts included “costs”, “abuse”, and “dependency”. On average, each participant added 1.43 positive concepts (SD = 1.35) and 0.94 negative concepts (SD = 1.31), indicating a slight overall positive evaluation of soft robots, compared to rigid ones. Notably, 33% of participants did not add any concepts that were evaluated negatively.

### 2.3 Data preparation

**FIGURE 5:**
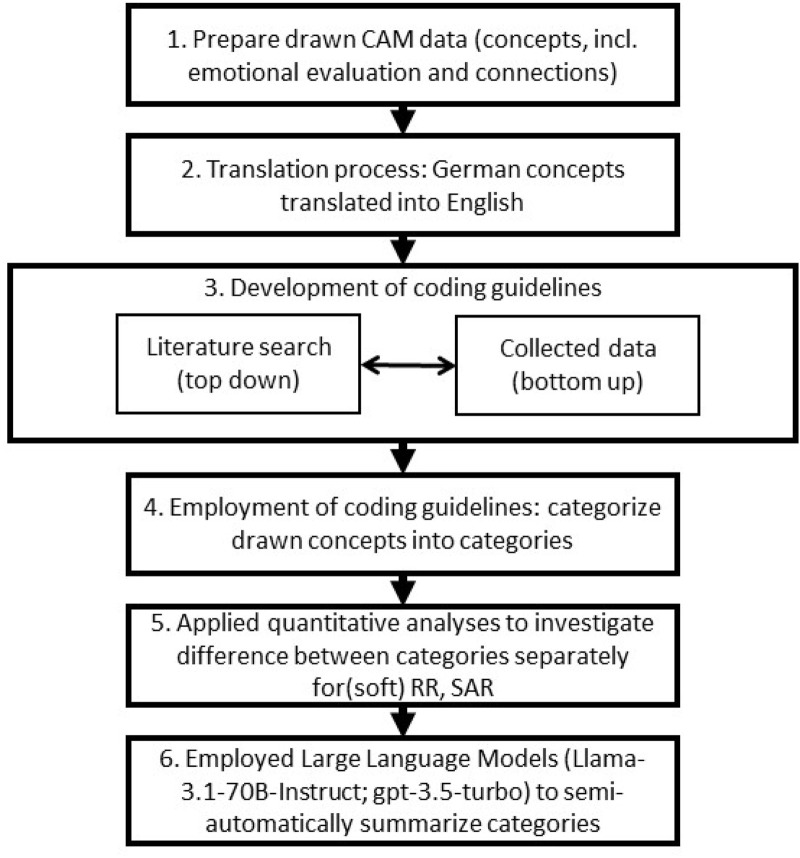
Illustration of the data preparation and analysis process involving a six-step procedure.

The data preparation and analysis followed a structured six-step procedure adapted from a previous CAM publication (Fenn et al., 2023) and qualitative content analysis (Kuckartz and Rädiker, 2022; Mayring, 2022). As illustrated in Figure 5, the process starts with the preparation of the drawn CAM data, including the extraction and cleaning of all concepts, and inter-concept connections. In a second step, all participant-generated concepts were translated from German into English. Subsequently, the research team developed detailed coding guidelines through a combined top-down and bottom-up approach: theoretical categories were first derived from the literature on human–robot interaction (e.g., Bröhl et al., 2019) and technology acceptance (e.g., Heerink et al., 2010), and then refined inductively based on recurring patterns observed in the CAM data. These coding guidelines enabled a transparent and reproducible categorization of all concepts into meaningful risk- and benefit-related dimensions. In the fourth step, the finalized coding guidelines were systematically applied to the entire dataset by multiple coders to classify each concept into its corresponding category. Building on this coded dataset, quantitative analyses were conducted to examine differences in category frequencies between robot types (SAR, RR) and across their rigid and soft embodiments. In the final step, Large Language Models (Llama-3.1-70B-Instruct; GPT-3.5-Turbo) were employed to semi-automatically summarize argument structures within each category (Hämäläinen et al., 2024). The automatically generated summaries and coding suggestions were subsequently reviewed and validated by the first two authors to ensure conceptual accuracy, consistency, and interpretive reliability. This integrated workflow ensured a systematic, transparent, and replicable analysis of both cognitive and affective dimensions in participants’ representations of soft and rigid robots.

### 2.4 Technical aspects

The analyses were performed using the statistical software R (R Core Team, 2020) and Python (Van Rossum and Drake, 2009), employing tools such as the rmarkdown package (Xie et al., 2018) and Jupyter Notebook (Kluyver et al., 2016) to produce reproducible dynamic scripts. These dynamic scripts integrate code, output, and text within a single document, facilitating the transfer of the proposed methodology to future studies. R was utilized to conduct quantitative analyses and generate visualizations, leveraging packages like psych (Revelle, 2021) and ggplot2 (Wickham, 2016). Python, on the other hand, was primarily used to apply the OpenAI API alongside LangChain for creating textual summaries of the specific categories. Detailed information about the applied packages (in R) and modules (in Python) can be found in the analysis files available on GitHub (https://github.com/FennStatistics/Article_SoftRobotIntervention).

The study was conducted online using the participant marketplace Prolific. Participants were required to be fluent in German and reside in Germany. Throughout both studies, we adhered to established standards of web-based research (Reips, 2021; Sauter et al., 2020). The studies were programmed using lab. js (Henninger et al., 2022), which provided the flexibility to collect paradata, such as recording when participants exited the full screen mode during the online study. The studies were hosted on a local university JATOS server (Lange et al., 2015), ensuring the highest privacy standards were maintained.

## 3 Results

This study systematically evaluated contrastive evaluations (pre-intervention vs. post-revision) of rigid and soft SARs and RRs through a combination of quantitative and qualitative analyses, focusing on emotional evaluations and the underlying argument structures for perceived risks, safety, and human-robot interaction. Our findings highlight nuanced differences in participants’ evaluations: Quantitative results demonstrated significantly more positive emotional evaluations and an increased number of drawn concepts after the intervention. Qualitative analyses revealed distinct argument structures and shifts in conceptualizations. Using a three-step analysis procedure, we first mapped participants’ pre- and post-intervention drawn concepts into key categories such as perceived safety, trust, and human-robot interaction (positive and negative), uncovering category-specific differences between rigid and soft robots. Second, Large Language Models (LLMs) identified shared and distinct argument structures across robot types, with rigid robots associated with precision and operational reliability and soft robots linked to emotional engagement and adaptability. Finally, detailed coding revealed distinct differences in perceived safety, risks, and the nature of the human-robot Interaction. For example, post-intervention CAMs—revised after participants read the soft-robot scenarios—contained more references to robots as safe and emotionally engaging, while also introducing concerns such as fragility and emotional dependency. By contrast, pre-intervention CAMs, referring to the rigid-robot scenarios, more frequently emphasized reliability and precision and highlighted concerns about technical issues and emotional detachment.

### 3.1 Quantitative analyses of perceived risks and benefits

**FIGURE 6:**
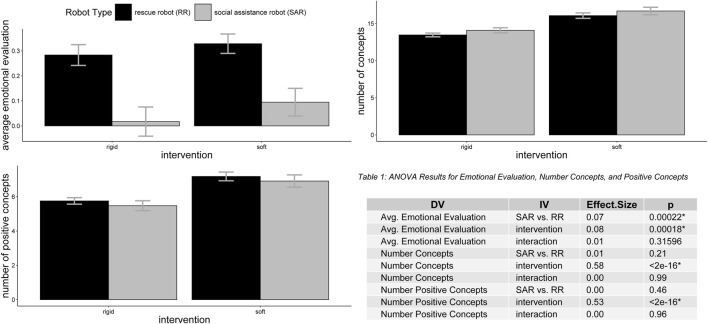
Comparison of emotional evaluation, and drawn concepts between rigid and soft SAR & RR shows the differences of the rigid (pre) and soft (post) condition differentiated by RR and SAR for the average emotional evaluation (top left), number of drawn concepts (top right), and the number of drawn positive concepts (bottom left). Note: the legend shown in the top-left graphic applies to all graphics.

The quantitative results indicate that both robot types, emotional evaluations became more positive in post-revision CAMs (after the soft-robot scenario) than in pre-intervention CAMs (after the rigid-robot scenario). In addition, the RR and SAR were perceived differently, with the RR eliciting a significantly more positive emotional evaluation. Figure 6, provide an overview of the effects of robot type (SAR & RR) and the intervention on three key outcomes: emotional evaluation, the total number of drawn concepts, and the number of negatively rated concepts.

The analysis revealed that the type of robot significantly influenced the average emotional evaluation of drawn concepts, with a medium effect size, F(1,178)=14.27 , p<.001 , $\eta_{\text{partial}}^{2}=.074$ . Emotional evaluations were significantly more positive after the intervention, F(1,178)=14.68 , p<.001 , $\eta_{\text{partial}}^{2}=.076$ , while the interaction between robot type and intervention was not significant, indicating consistent patterns of change across robot types, F(1,178)=1.01 , p=.316 .

The number of drawn concepts did not differ significantly by robot type, F(1,178)=1.60 , p=.208 , $\eta_{\text{partial}}^{2}=.009$ , but the intervention showed a large main effect, F(1,178)=250.05 , p<.001 , $\eta_{\text{partial}}^{2}=.584$ , reflecting substantially more drawn concepts after the intervention. This trend persisted uniformly across robot types, as evidenced by a non-significant interaction effect, F(1,178)<0.01 , p=.989 .

For positively rated concepts, the results indicated that robot type had no significant effect, F(1,178)=0.54 , p=.462 , $\eta_{\text{partial}}^{2}=.003$ , while the intervention exhibited a very large and highly significant effect, F(1,178)=200.33 , p<.001 , $\eta_{\text{partial}}^{2}=.530$ . The interaction between robot type and intervention remained non-significant, F(1,178)=0.00 , p=.959 , suggesting that the changes over time occurred similarly regardless of robot type.

In the next section, we analyze the underlying argument structures to examine which reasons participants emphasized when revising their CAMs. Given the sequential exposure (rigid-robot scenario first, soft counterpart second) and the instruction to revise an previously drawn CAM, the qualitative patterns reported below are interpreted as contrastive emphases elicited under explicit comparison, rather than as exhaustive belief representations about each robot embodiment in isolation.

### 3.2 Qualitative analyses of perceived risks and benefits

**FIGURE 7:**
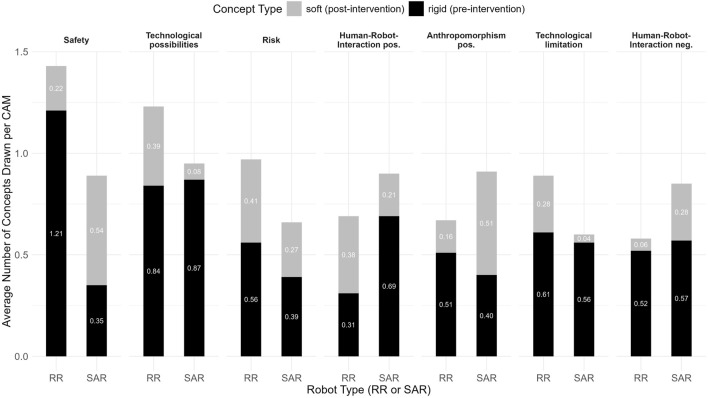
Stacked bar chart of drawn rigid and soft concepts by robot type presents a breakdown of participants’ average number of drawn concepts per CAM, categorized by rigid (“constant”) and soft (“new”) concepts. The stacked bars highlight how participants retained existing ideas (rigid) or introduced new concepts (soft) in response to the intervention.

**FIGURE 8:**
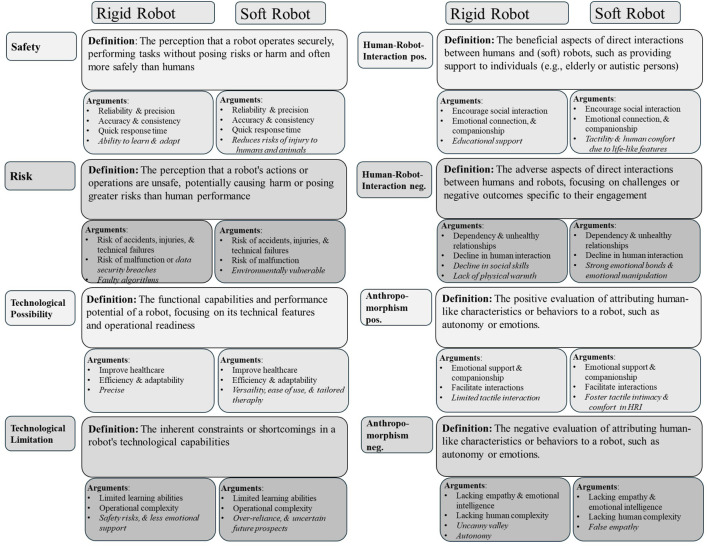
Bubble graph of overall argument structures presents a graphical synthesis of the most significant qualitative arguments, distinguishing between rigid and soft robots, alongside definitions for the coding guideline categories (see for detail “4. Coding guidelines categories” in the Supplementary Material). Shared perceptions are represented by overlapping bullet points, while differences in argument structures are highlighted in italics.

**FIGURE 9:**
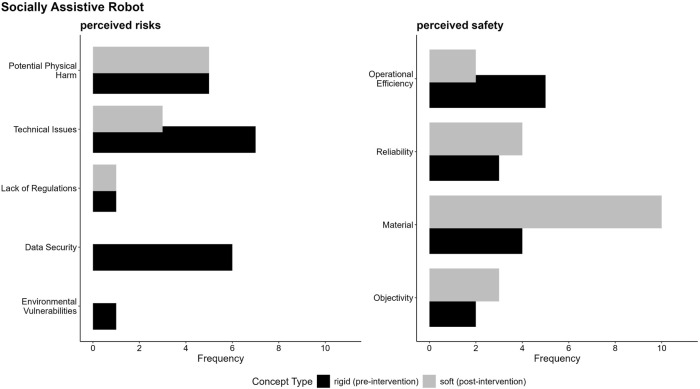
Argument frequencies on perceived risks and safety of rigid and soft SAR depicts key arguments related to the perceived risks and safety. Argument frequencies highlight how often a single argument (code) was discovered within the respective category. Detailed coding guidelines and descriptions of individual codes are available in “5. Coding guidelines codes (within categories)” in the Supplementary Material.

**FIGURE 10:**
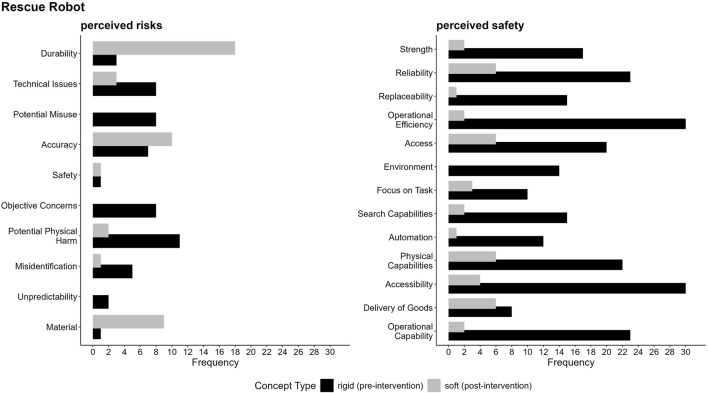
Argument frequencies on perceived risks and safety of rigid and soft RR depicts key arguments related to the perceived risks and safety.

**FIGURE 11:**
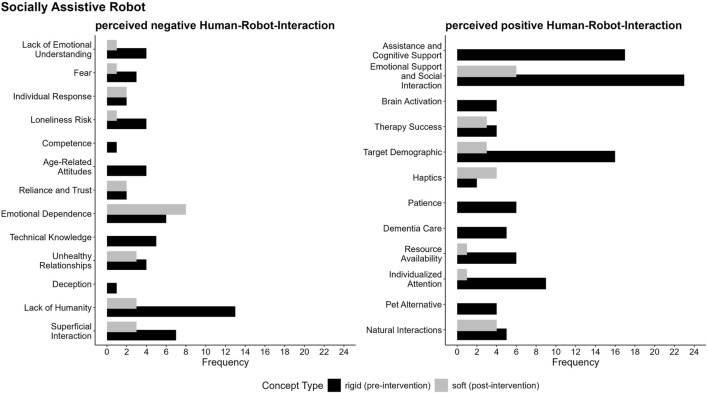
Argument frequencies on perceived positive and negative human-robot interaction of rigid and soft SAR illustrates key arguments regarding both the benefits and challenges associated with human-robot interaction.

**FIGURE 12:**
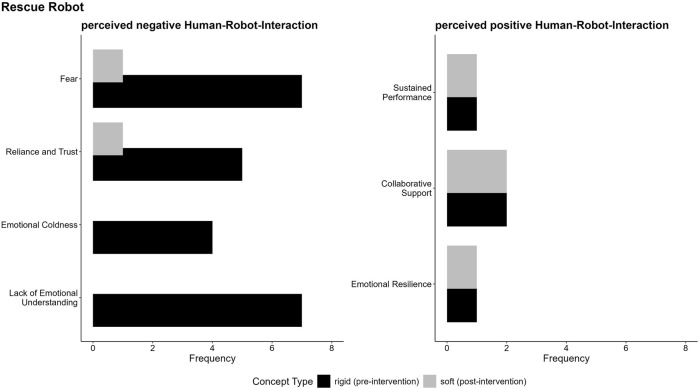
Argument frequencies on perceived positive and negative human-robot interaction of rigid and soft RR illustrates key arguments regarding both the benefits and challenges associated with human-robot interaction.

To analyze participants’ perceptions of rigid and soft SAR and RR, we conducted a three-step qualitative analysis, which should be interpreted as structured aggregations of expressed contrastive arguments. First, we systematically categorized the CAM data using a primarily deductive qualitative content analysis, involving six independent raters, to map participants’ conceptualizations before and after the intervention, focusing on key categories (e.g., perceived safety), as visualized in a stacked bar chart (Figure 7). Second, we applied LLMs to inductively analyze argument structures, highlighting shared and distinct perceptions of rigid and soft robots, depicted in a bubble graph (Figure 8). Finally, we examined the frequency and distribution of argument structures within the identified categories to uncover shifts in participants’ evaluations, summarized through detailed code-specific visualizations based on a mainly inductive driven qualitative content analysis (Figures 9–12). The coding guidelines for the two qualitative content analyses are accessible in the Supplementary Material. A reproducible script, including the exact prompting of the LLMs, is available in the “main study - LLM” folder on GitHub.

#### 3.2.1 Systematic categorization of key categories

In the first step of the qualitative analysis, we systematically coded all CAM data to map participants’ conceptualizations of rigid and soft SAR and RR across core categories such as “safety”, “risk”, “trust”, “mistrust”, “human-robot interaction positive”, “human-robot interaction negative”, “social impact positive”, “social impact negative”. Visualizing the frequencies of these categories as a stacked bar chart (Figure 7) revealed category-specific differences between rigid and soft RR and SAR. For example, the perceived safety (SA) category was most important for the RR, where on average 1.4 concepts were drawn. Further, the perceived risks (R) were more important for the RR and participants mentioned more technological possibilities. In contrast for the SAR, categories regarding the perceived positive and negative Human-Robot-Interaction (HRIP, respectively HRIN) and perceived negative anthropomorphism (AN) were more frequently mentioned in comparison to the RR. Additionally, for SAR more concepts after the intervention (soft) were added for the SA and positive anthropomorphism (AP) categories, underscoring their relevance to this type of robot.

#### 3.2.2 Overall argument structures

The findings illustrated in Figure 7 highlight participants’ context-dependent evaluations of SAR and RR. While some categories overlap, significant distinctions are evident. SARs were primarily evaluated in terms of their social interaction capabilities, RRs maintained stable associations with safety and operational reliability, highlighting the distinct roles and perceived risks and benefits of these robot types across different scenarios. This motivated us to apply LLMs, to comprehensively synthesize the full dataset and to identify key argument structures across the most relevant categories, as visualized in Figure 8, whereby different overarching themes as well as critical distinctions between the two robot types emerged. A deeper examination of participants’ comments revealed their underlying argument structures. In participants’ pre-intervention CAMs, rigid robots were described as technologically advanced and precise but are often criticized for lacking emotional engagement and physical safety. For example, one participant (P3) observed that “soft robots may not be as precise as other rescue robots or humans, which is a disadvantage.” In contrast, in their post-intervention CAMs, participants described soft robots as safer in direct human–robot interactions and emphasized adaptability and closer human connections. At the same time, concerns about over-dependence, emotional vulnerability, and environmental robustness persist (see in detail Figure 9). Regarding the perceived safety, one participant (P4) noted that “soft robots pose only a minimal risk to the victims due to their flexibility”, which might be an advantage during search and rescue missions. For soft SAR on the other hand, some participants expressed the fear that soft robots might lead to “emotional dependency”, which could result in a loss of social contact and social skills.

#### 3.2.3 Category-specific argument frequencies and structures

The findings in Figure 8 highlight the nuanced and context-specific perceptions of robot types, which is further explored in the following category specific analysis for the categories perceived safety and risks, as well as the perceived positive and negative human-robot-interaction. To better understand these perceptions, we used LLMs to (a) inductively code the data within each category and (b) produce a context-sensitive summary of participants’ evaluations. This method also considered the frequency of single arguments as an indicator of their relative importance, alongside the developed code descriptions and the marked text passages identified in step (a). The analysis reveals key distinctions and overlaps in argument structures, helping us uncover the factors that shape perceptions within each category. Additionally, to visually represent the distribution and frequency of arguments, we created code-specific visualizations (see Figures 9–12), facilitating a clearer comparison of participants’ evaluations for rigid and soft robots across perceived positive and negative aspects of risks and safety concerns and human-robot interactions. In the subsequent sections, we explain the specific argument structures for each category—perceived safety, risks, and human-robot interaction—separately for SAR and RR, with detailed descriptions provided below the respective figures.

##### 3.2.3.1 Perceived safety and risks

In the following paragraph we outline the safety and risk concerns for SAR and RR. Based on this, we derived design recommendations in the discussion (see Table 1).

A primary concern as shown in Figure 9 regarding the perceived risks is the potential for physical harm due to rigid and soft robots, with participants expressing concerns about malfunctioning robots causing unintended reactions or injuries. Technical issues, such as malfunctions and glitches, are also a significant concern particularly for rigid robots, which are seen as more prone to errors and accidents. The risk of data security breaches is another prominent concern, primarily associated with rigid robots, as participants worry about the potential for data losses and misuse. In contrast, soft robots are perceived as more vulnerable to technical problems due to their limited technical abilities which could lead to helplessness in case of failures. Overall, the findings highlight the importance of data security, mitigating technical issues and taking concerns including the prevention of unintended reactions or injuries seriously to ensure the safe and reliable integration of socially assistive robots into human environments.

When assessing the perceived safety of SAR, participants frequently referred to material composition, with most such references emerging after the soft-robot intervention and linking safety explicitly to material softness. Participants frequently described softer materials as potentially reducing the risk of injury during interactions or accidents. This pattern is consistent with the information provided in the soft-robot scenario and therefore likely reflects participants’ incorporation of scenario content rather than an emergent, independent association (see Supplemental Data). This perception of material safety was closely linked to the reliability of both rigid and soft robots, which were frequently characterized as dependable systems capable of continuous operation, rapid emergency responses, and precise decision-making. Furthermore, the objectivity of robots, attributed to their lack of emotional bias, was identified as a significant advantage, particularly in contexts requiring impartial decision-making. Operational efficiency emerged as a notable strength of rigid and soft robots, given their ability to perform tasks with greater speed and precision. Overall, the findings underscore that the perceived safety benefits of SAR—particularly soft robots—stem from their reliability, impartiality, and physical safety, which help foster trust and encourage broader adoption across various applications.

Regarding the perceived risks of RR, durability, and material defects are key concerns for soft robots, with participants frequently emphasizing their fragility and vulnerability to damage in unpredictable environments as shown in Figure 10. Conversely, rigid robots are more often associated with technical issues, potential misuse, potential physical harm during rescue missions, and broader concerns such as developmental errors. Both types of robots raise concerns about accuracy, particularly the risk of imprecision or performance limitations under extreme conditions. This underscores the need for robust mitigation strategies to ensure their reliable deployment in rescue scenarios.

The primary benefit of rescue robots regarding their perceived safety lies in their operational efficiency, allowing for improved performance, reduced response times, and safer interventions that minimize risks for both victims and rescuers. Reliability was also emphasized, with participants valuing consistent performance, precise control, and dependable operation under hazardous conditions. Physical capabilities, such as the ability to lift heavy objects, navigate challenging terrains, and perform tasks beyond human limitations, were highlighted as critical attributes. Accessibility, particularly in confined or hard-to-reach spaces, further enhanced their perceived utility and safety. While rigid robots were noted for their strength and reliability, soft robots were appreciated for their adaptability, flexibility, and reduced risk of causing injury during rescue operations. Additionally, their ability to deliver goods and access remote locations was identified as a key benefit. Overall, the perceived safety of rescue robots is strongly associated with their capacity to operate effectively in hazardous environments, safeguard human lives, and maintain reliable and efficient performance during rescue operations.

##### 3.2.3.2 Perceived positive and negative human-robot-interaction

The following paragraph outlines the perceived positive and negative human-robot-interaction for the SAR and RR. Based on this, we also derived design recommendations in the discussion (see Table 1).

A key concern of the perceived risks of human-robot interaction with SARs is the perceived lack of humanity in human-robot interaction (see Figure 11). Participants emphasized the irreplaceable value of human empathy and authentic interactions, especially with rigid robots. This concern is closely linked to the risk of emotional dependence, which participants identified as a significant issue especially for soft SARs, raising fears of individuals forming strong, potentially unhealthy attachments. Additionally, apprehensions about superficial interactions, loneliness, deceptive behaviors, and the potential for unhealthy relationships highlight the complexity of emotional dynamics in human-robot interaction. Rigid robots were perceived as more intimidating and potentially fear-inducing, with a lack of emotional understanding, while soft robots sparked concerns about emotional dependency and trustworthiness. Technical knowledge and competence were also emphasized, particularly for rigid robots, as essential for safe and effective use. Overall, the findings indicate that the successful integration of socially assistive robots requires balancing emotional understanding and empathy to address risks like emotional dependency and the lack of humanity, which could hinder acceptance and effectiveness.

The perceived benefits in human-robot interaction are diverse, with participants emphasizing SARs’ potential to provide emotional support and foster social interaction, particularly for individuals experiencing loneliness or isolation. Robots were noted for their ability to enhance engagement, offer companionship, help in therapeutic settings and address social isolation, making them particularly valuable for socially isolated individuals, older adults, and those with special needs or learning difficulties. Additionally, participants highlighted the capacity of robots to improve efficiency, and support individuals with depression by providing consistent attention and care. Due to their soft haptics, soft robots were perceived as offering unique advantages, such as their capacity to foster emotional attachment and simulate a realistic “companion”, contributing to therapeutic outcomes. Features like soft materials and tactile interactions were seen as improving acceptance and comfort, enhancing the overall experience of human-robot interaction. Overall, the benefits of socially assistive robots lie in their ability to deliver individualized attention, emotional support, and patience, with soft robots uniquely positioned to facilitate more natural and empathetic interactions.

In comparison to the SAR less arguments were mentioned (compare Figures 11, 12). In the context of human-robot interaction for RR, negative perceptions were largely influenced by fear of the robots, concerns about their reliance and trustworthiness, and the perceived lack of emotional understanding. The most significant apprehensions centered around the fear and discomfort caused by emotionally detached robots, with participants emphasizing the critical need for emotional understanding and empathy in rescue scenarios. This concern was particularly pronounced for rigid robots, which were often described as emotionally cold, lacking humanity, and incapable of recognizing or responding to distress. These limitations were seen as significant barriers to their effectiveness in providing emotional support during emergencies. In contrast, such concerns were only rarely associated with soft robots. Overall, the findings highlight the need to address emotional and social dimensions in the design of rescue robots, with soft robots offering promising potential to better meet these requirements.

In the context of positive perceptions of human-robot interaction for rescue robots, participants provided only a few arguments. Both rigid and soft robots were recognized for their potential to offer collaborative support, enhancing human capabilities through partnership rather than replacement. Sustained performance also emerged as a key consideration, highlighting the importance of maintaining physical and operational endurance during extended rescue missions. Notably, the findings suggest that soft robots, with their potential to foster collaborative support, ensure sustained performance, and provide emotional reassurance, may offer a more comprehensive and effective solution in rescue operations, benefiting both victims and rescue teams.

### 3.3 Exploratory analysis of gender differences in perceived risks and benefits of human-robot interaction for SAR

In this exploratory analysis, because of limited sample size, we aimed to illustrate the capability of our methodology to identify only gender-related differences in perceptions of human-robot interaction. To demonstrate this approach, we focused on highlighting key differences between participants identifying as men and women respectively in their perceptions of the positive and negative aspects of human-robot interaction as an exemplary case.

For example, for human-robot-interaction, both genders value the utility of SARs in providing emotional support, individualized care, and cognitive assistance, particularly for older or socially isolated individuals. Shared emphasis is placed on features like haptics and soft materials for fostering acceptance and the robot’s patience, which is deemed critical in tasks such as dementia care. However, women prioritized emotional connection and companionship, framing robots as potential alternatives to pets and focusing on reducing shame and supporting “touch-starved” individuals. In contrast, men emphasized natural interactions and functional utility, such as overcoming linguistic barriers, enhancing therapeutic applications, and improving social competence. For risks, both genders expressed concerns about emotional dependence and the inability of robots to replace meaningful human interactions. Women highlighted the emotional impacts of robot failures and diverse comfort levels with physical and emotional engagement. Men, however, focused more on ethical concerns, such as deception, the superficiality of robot interactions, and potential degradation of human social behavior due to dependency.

## 4 Discussion

This study systematically evaluated participants’ contrastive evaluations (pre-intervention vs. post-revision) of rigid and soft SARs and RRs through quantitative and qualitative analyses. In doing so, we highlighted key differences in the emotional evaluation and the argument structures behind these two application cases. Quantitative results revealed a significantly more positive evaluation and an increased number of drawn concepts after participants revised their previously drawn CAM after being informed about the potential risks and benefits of transitioning from rigid to soft robots. Qualitative findings disentangled the argument structures participants emphasized when revising their CAMs separately for the two types of robots and revealed key distinctions in how participants conceptualized the perceived risks and benefits of rigid and soft SARs and RRs. To achieve this, a three-step qualitative analysis was conducted. First, the CAM data were systematically categorized through a primarily deductive qualitative content analysis, mapping participants’ conceptualizations before and after the intervention across core categories such as perceived safety and human-robot interaction. Second, LLMs were employed for an analysis of argument structures, identifying shared and unique perceptions of the robot types, which were visualized in a bubble graph. Finally, shifts in argument structures and their frequencies within categories were examined by applying an inductive qualitative content analysis. These findings were visually represented and summarized in a data-driven manner, focusing on the categories of perceived risks and safety as well as positive and negative aspects of human-robot interaction. Together, these analyses provide a structured summary of revision-based, contrastive evaluations of participants’ belief structures regarding the perceived risks and benefits of rigid versus soft SARs and RRs.

Furthermore, our study design enables the investigation of (soft) robots still in development, thereby allowing for the collection of larger sample sizes compared to traditional laboratory studies (Whelan et al., 2018). Such studies will enable the further investigation of perceived risks and benefits across various applications of both soft and rigid robots and enhance existing design evaluation tools, such as Exoscore, by uncovering factors that predefined questionnaires might overlook (Shore et al., 2020). However, it is important to note that these online studies should be complemented by in-lab studies, which remain crucial for validating findings and exploring specific interactions in controlled settings, especially when fully developed prototypes are available or when more in-depth qualitative data is needed. However, due to the scenario-based approach, our study design enables early identification of societal concerns, which are crucial knowledge during the robotic design process (Šabanović et al., 2023). By addressing these concerns early in the development, rapid adjustments in robot design might be possible. Such an approach can avoid a misalignment of opinion between end users and developers on desirable design features (Bradwell et al., 2019). Further, by including different age groups, design preferences between older and younger people can be discovered (Chu et al., 2019; Piezzo and Suzuki, 2017). In Table 1, we present participant-derived considerations, translating these contrastively elicited considerations into design recommendations for early development, which could bridge the gap between user perceptions and actionable design criteria, enabling developers to address specific concerns effectively.

### 4.1 Limitations

A central limitation of this study concerns the interpretation of changes between pre- and post-intervention CAMs. Because we employed a single-group pre–post design, the observed differences between the “rigid” and “soft” conditions cannot be taken as causal evidence that particular benefits are intrinsic features of soft robotics. These differences may instead reflect a combination of (a) intrinsic properties of soft robotics that participants find appealing, (b) information artifacts, such as participants echoing scenario content, and (c) temporal effects, including elaboration over time independent of robot type. This revision-based procedure also raises established methodological considerations (Baxter et al., 2016; Bethel and Murphy, 2010; Hoffman and Zhao, 2020; Rosenthal-von der Pütten et al., 2025): First, instructing participants to revise a previously drawn CAM can introduce demand characteristics, insofar as visible modification becomes a salient cue of task compliance. Second, the measurement activity itself (drawing and revising CAMs) may induce reactive measurement effects and repeated-measurement elaboration (learning/habituation), such that participants generate additional concepts or adjust valence partly due to reflection and the structure of the task. Third, presenting the rigid scenario first may create an anchoring point for subsequent revisions, such that post-revision content reflects anchoring-and-adjustment around an initial representation rather than anew belief construction. As such observed quantitative shifts (e.g., higher mean valence and increased number of drawn concept after post-revision) cannot be interpreted as standalone psychological effects of robot embodiment, they may be amplified by repeated measurement, compliance cues, and elaboration over time.

These design related limitations interact closely with the provided scenario texts, making the framing a second, closely related limitation: This is particularly the case for SAR, whereby we unintentionally emphasized special-needs applications, which may have skewed participants’ evaluations toward more positive assessments. While the within-subject comparison reduces between-participant variance by having the same participants complete both conditions, this only partially mitigates the limitation, as it does not remove scenario- or order-dependence, and fixed sequential exposure can still introduce, for example, anchoring, or elaboration effects. Beyond these interpretive (internal-validity) constraints, the study also has scope-related limitations that affect generalizability: Future research should explore a broader range of scenarios to capture diverse contexts of application. Another limitation stems from the study’s limited geographical scope, as the sample was drawn exclusively from Germany. This constraint restricts the ability to generalize findings to other cultural contexts. Extending the study to a multi-country design, considering diverse cultural and value systems, would help to broaden the applicability of the results and offer insights into how different cultural contexts influence the perception of soft robots.

Several design refinements could increase confidence in which effects extend beyond the within-subject contrastive procedure: (i) counterbalancing/rotating the order of the presented rigid, soft robot embodiment to quantify carryover, learning, or novelty effects, (ii) instructing participants to construct the second CAM from scratch (followed by an optional comparison step) to reduce anchoring (explicit comparison), (iii) adding a between-subjects CAM condition (rigid-only vs. soft-only) as a robustness check to isolate differences that persist without explicit comparison.

### 4.2 Conclusion

Involving laypersons in the early phases of robot design provides valuable insights into everyday reasoning, enabling the identification of social, ethical, and practical concerns that may otherwise remain unnoticed during purely technical development. The present study demonstrates how our intervention-based methodology, combining Cognitive-Affective Mapping with quantitative and LLM-supported qualitative analyses, offers a systematic and scalable approach for eliciting and structuring such user perspectives. By organizing perceptions into key categories—such as perceived risks, safety, trust, and human–robot interaction dynamics—this approach facilitates the integration of user-centered feedback into early design iterations, fostering robotic systems that are both functionally robust and socially acceptable (Pinskier and Howard, 2022; Stella and Hughes, 2023). Recognizing the complexity and ethical implications of deploying robots in human-centered and safety-critical domains such as socially assistive and search-and-rescue robotics (Chitikena et al., 2023; Ward et al., 2014), our study highlights the potential of early, low-cost, and scalable engagement methods for responsible innovation. By identifying and systematically analyzing the contrastive argument structures underlying public perceptions, developers and policymakers can proactively address user concerns, reduce misalignments between societal expectations and technological design, and ultimately promote a more transparent and ethically grounded development of soft robotic systems.

## Data Availability

All data, code, and materials used in the analysis are available on GitHub (https://github.com/FennStatistics/Article_SoftRobotIntervention). Software to create the Cognitive-Affective Maps can be accessed via https://drawyourminds.de/.
