# Supplementary material for: Informing robot design through early public engagement: lay perceptions of soft versus rigid socially assistive and rescue robots
Source: Front Robot AI. 2026 Mar 19;13:1741946. doi: 10.3389/frobt.2026.1741946 (PMC13043413; doi:10.3389/frobt.2026.1741946)
Supplement: Supplementary file 1 [file Supplementaryfile1.pdf]

Supplementary Material for Article ”Informing Robot Design through Early Public Engagement: Lay Perceptions of Soft versus Rigid Socially Assistive and Rescue Robots”

1 FEEDBACK EXPERTS

Experts of the Cluster of Excellence Living, Adaptive and Energy-autonomous Materials Systems (*liv*MatS) were asked to answer six questions regarding the risks and benefits of soft robots compared to rigid robots. They were encouraged to focus on application cases, ethical considerations such as safety, privacy, societal impact, and sustainability, providing concise expert insights into the most significant risks and benefits for each case study. In the following, a concise bullet-point list based on the responses to each question is presented separately:

Q 1: What are the most promising applications of soft robots over their rigid counterparts within your field of expertise?

- Enhanced adaptability and ability to fit into tight or inaccessible spaces, including harsh or hazardous environments (e.g., marine, mines, radioactive sites).
- Applications in medical fields such as handling sensitive objects, rehabilitation, and soft exoskeletons.
- Agriculture and industry: manipulation of fragile objects, fruit harvesting, and animal handling.
- Safer human-machine interaction, including social care and increased acceptance due to reduced risk of injury.
- Use in areas requiring electronic-free solutions, such as explosive or high-EM environments (e.g., war zones, nuclear plants).

Q 2: In your expert opinion, what are the main benefits of soft robots compared to rigid robots?

- Safer interaction with humans and the living world due to softer materials, reducing the risk of injury.
- Enhanced adaptability to diverse environments, conditions, and tasks, including harsh or unknown scenarios.
- Capability to handle fragile or sensitive objects with minimal damage, such as in agriculture or human care.
- Increased flexibility, lower energy use, and potentially lower costs compared to rigid robots.
- Broader application potential, including electronic-free designs suited for environments where sparks or electrical failures are a risk.

Q 3: In your expert opinion, what are the main risks of soft robots compared to rigid robots?

- Limited robustness and durability, with increased susceptibility to damage and shorter lifespan.
- Slower response times and restricted functionality compared to rigid robots.

- Challenges in repair, replacement, and load-carrying capacity, making them less adaptable to extreme conditions.
- Potential risks in specific environments, such as chemical exposure or harsh physical conditions.
- Ethical or practical concerns regarding the allocation of resources to potentially less promising research areas.

Q 4: From your expert perspective, what do you identify as the primary social benefits of soft robots compared to rigid robots?

- Safer interaction with humans due to less dangerous, flexible structures.
- Increased acceptance and familiarity due to life-like shapes and more natural appearances.
- Potential for greater human interaction and usability in everyday tasks.
- Energy and resource efficiency, contributing to sustainable applications.
- Long-term impact on shaping future robotics technology through innovative design.

Q 5: From your expert perspective, what do you identify as the primary social risks of soft robots compared to rigid robots?

- Poor design could lead to discomfort or fear, such as overly human-like or unappealing appearances.
- Misallocation of resources, with concerns about overfunding a niche field with limited market impact.
- Potential public rejection due to unrealistic expectations or lack of clear societal value.
- Ethical concerns about robots becoming overly human-like, potentially causing social unease.
- Overexposure to human interaction without sufficient utility, leading to diminished acceptance or trust.

Q 6: From your expert point of view, could you elaborate on the potential environmental impacts of soft robots, considering aspects like energy efficiency, and end-of-life disposal, particularly in comparison to rigid robots?

- Lightweight materials may lower energy consumption during the robot's lifespan.
- Potential for increased sustainability through the use of renewable or biodegradable materials and reduced reliance on rare earths or electronics.
- Longer lifespan due to better durability against physical damage, reducing waste.
- Complex manufacturing processes and difficulties in recycling soft robots currently pose environmental challenges.
- Energy efficiency is potentially better than rigid robots, but current pneumatic and hydraulic systems may still lag behind electrically powered alternatives in terms of overall efficiency

2 SCENARIO TEXTS

The following two scenario descriptions were provided to participants, depending on their random assignment to the socially assistive robots or rescue robot condition (between conditions). The scenario texts were presented on three separate pages indicated in the text.

2.1 Scenario Text: Socially Assistive (Soft) Robot

—————— First Page in Experiment ——————

Please read the following text carefully.

Afterwards we will ask you to draw a CAM around the predefined knotpoint:

"Which risks and benefits come to your mind when considering the use of socially assistive robots (SAR) for therapy, care for older adults, education or as social companions?"

Socially assistive robots are increasingly used in social assistive tasks such as therapy, care for older adults, education, or as social companions. They are designed to support human users through social interaction. The goal of socially assistive robots is to create close connections and effective interactions with human users in order to support rehabilitation, enhance learning, or offer companionship to those who are isolated. Since socially assistive robots are still in the development phase, it is important to consider the ethical aspects (= benefits and risks) of socially assistive robots.

—————————— Second Page in Experiment ——————————

Socially Assistive Robots

Possible benefits of socially assistive robots could include:

- Interaction with socially isolated or less socially connected individuals, such as older adults
- Promotion of social interaction in therapeutic settings, for example, as companions for people with autism to improve emotion recognition and interpersonal communication skills
- Support in educational activities and learning, especially for children with special needs or learning difficulties

Possible risks of socially assistive robots could include:

- Users might develop a dependency on socially assistive robots, especially if they provide significant support or help with daily activities
- The use of social assistive robots could lead to unemployment, as robots might replace people in the workplace (e.g., as therapists)
- Human-robot interactions could influence human interactions, as dealing with robots might shape our expectations, behaviors, and perceptions in social environments

—————————— Third Page in Experiment ——————————

The development of socially assistive robots is still in its early stages. You can contribute to the development of ethically safe socially assistive robots. For this purpose, we would like to find out your attitudes and feelings towards socially assistive robots. To this end, we have predefined the central concepts "socially assistive robot," "risks," and "benefits" at the center of your CAM.

From these given concepts, only the emotional evaluation and not the text can be changed. We ask you to draw your thoughts and feelings regarding the question *"Which risks and benefits come to your mind when considering the use of socially assistive robots (SAR) for therapy, care for older adults, education or as social companions?"* in your mind map. For this, you should draw all the benefits and risks that come to mind regarding socially assistive robots around the given concepts "socially assistive robot," "benefits," and "risks."

——————— First Page in Experiment (Soft Robot) ———————

Please read the following information on soft robots carefully. Afterwards we will ask you to adjust your CAM.

Currently, there is a trend towards using so-called soft robots for socially assistive tasks. Soft robots are a novel class of robots often inspired by the characteristics of living organisms, such as animals. Unlike other robots typically made from hard materials like metal or hard plastic, soft robots usually contain no electronic parts and are made from flexible, soft materials such as silicone. They often take natural forms and can bend, twist, and stretch, similar to living organisms, like snakes or octopuses.

——————— Second Page in Experiment (Soft Robot) ———————

Soft Socially Assistive Soft Robots

Possible benefits of socially assistive soft robots could include:

- Lower risk of injury during physical interactions due to their softness
- Promotion of social interaction in therapeutic settings, for example, as companions for people with autism to improve emotion recognition and interpersonal communication skills
- Natural and intuitive interaction with humans, as the soft structure and flexibility of soft social assistive robots can enable human-like movements and reactions

Possible risks of soft social assistive robots could include:

- Emotional dependency due to the lifelike characteristics of soft robots, which could potentially limit engagement in human interactions
- Human-robot interactions could influence human interactions, as dealing with robots might shape our expectations, behaviors, and perceptions in social environments
- The ability of soft social assistive robots to evoke emotional responses could be problematic if it aims to influence the behavior or decisions of users

——————— Third Page in Experiment (Soft Robot) ———————

After reading the information about socially assistive soft robots, we would now like to ask you to adjust your CAM. You can add new benefits and risks or delete already drawn concepts.

2.2 Scenario Text: Search and Rescue (Soft) Robot

——————————— First Page in Experiment ———————————

Please read the following text carefully.

Afterwards we will ask you to draw a CAM around the predefined knotpoint:

"Which risks and benefits come to your mind when considering the use of robots in search and rescue missions (SRs)"

Search and rescue robots are a new type of robot designed to search for and rescue people in disaster situations (earthquakes, collapsed buildings, contaminated areas, etc.). Search and rescue robots, such as drones and ground robots, can operate in dangerous and contaminated areas that would otherwise be

inaccessible to human rescuers. By performing tasks such as visual inspection of damaged structures, searching for victims, mapping the affected area, clearing debris, providing vital supplies, and autonomously assisting in the rescue of victims, these robots can improve the efficiency of rescue operations. Since search and rescue robots are still in the development phase, it is important to consider the ethical aspects (= benefits and risks) of search and rescue robots.

——————— Second Page in Experiment ———————

Search and Rescue Robots

Possible benefits of search and rescue robots could include:

- Access to areas that are unreachable or too dangerous for human rescuers
- Reliable performance, especially for tasks that require precision and accuracy
- Autonomous rescue capabilities that allow robots to locate and bring victims to safety

Possible risks of search and rescue robots could include:

- Algorithms guiding the rescue robots could be biased, leading to unfair or discriminatory outcomes, especially regarding where to focus rescue efforts and whom to search for and rescue first
- The level of autonomy of rescue robots in search and rescue operations could raise the question of whether remote control of robot operations in precarious situations is preferable to full autonomy
- Rescue robots could be misused, especially in warfare

——————— Third Page in Experiment ———————

The development of search and rescue robots is still in its early stages. You can contribute to the development of ethically safe robots for search and rescue missions. For this purpose, we would like to find out your attitudes and feelings towards rescue robots. To this end, we have predefined the central concepts ”search and rescue robots,” ”benefits,” and ”risks” at the center of your mind map. From these given concepts, only the emotional evaluation and not the text can be changed.

We ask you to draw your thoughts and feelings regarding the question *”Which risks and benefits come to your mind when considering the use of robots in search and rescue missions?”* in your CAM. For this, you should draw all the benefits and risks that come to mind regarding search and rescue robots around the given concepts ”search and rescue robots,” ”benefits,” and ”risks.”

————— First Page in Experiment (Soft Robot) —————

Please read the following information on soft robots carefully. Afterwards we will ask you to adjust your CAM.

Currently, there is a trend towards using a new type of so-called soft robots for search and rescue missions. Soft robots are a new kind of robot which are designed to mimic the properties of living entities such as animals. Unlike normal robots, which are typically composed of hard materials like metal or hard-plastic, soft robots do not have electronic devices in them and are made of flexible, soft materials like silicone. They often have natural shapes and can bend, twist, and stretch like living organisms, such as snakes or

octopi. Designed with inspiration from living entities, these soft robots often look and feel more lifelike than rigid robots.

——————— Second Page in Experiment (Soft Robot) ———————

Soft Robots for Search and Rescue Missions

Possible benefits of soft robots for search and rescue missions could include:

- Access to areas that are unreachable or too dangerous for human rescuers
- Delivery of vital supplies (water, food, medicine) until victims are safely rescued
- Reduction of injury risk for victims due to their flexibility and adaptability

Possible risks of soft robots for search and rescue missions could include:

- The soft and adaptable nature of soft robots could potentially lead to damage to the robot in dangerous environments
- Algorithms guiding the soft rescue robots could be biased, leading to unfair or discriminatory outcomes, especially regarding where to focus rescue efforts and whom to search for and rescue first
- Due to their flexibility, soft rescue robots might be less precise in certain tasks that require high accuracy

——————— Third Page in Experiment (Soft Robot) ———————

After reading the information about soft rescue robots, we would now like to ask you to adjust your CAM. You can add new benefits and risks or delete already drawn concepts.

3 FEEDBACK ADAPTIVE QUESTION LAYPERSONS

Participants were asked to create two mind maps to explore their perceptions of a rigid and a soft socially assistive robot/ rescue robot. Each Cognitive-Affective Map (CAM) included an emotional rating for the concepts they associated with each type of robot. After completing both mind maps, participants were presented with an adaptive follow-up question to reflect on the emotional ratings of their CAMs. The question was tailored to their individual responses and framed as follows, where XXX represents the average emotional rating from their drawn CAMs:

> ”Your adapted mind map had an average emotional rating of XXX, which was [more negative/more positive] compared to your initially drawn mind map (average emotional rating of XXX). Please explain why you perceived this [more negative/more positive].”

In the following a concise bullet-point list based on the responses to this question is presented separately for the socially assistive robot and rescue robot, whereby only responses are considered where the average emotional rating of the post CAM was more negative (1 SD below the average) or more positive (1 SD above the average):

Socially Assistive Robot, negative post CAM

- **Ethical Concerns and Potential Misuse**
  Participants expressed concerns about unethical behavior, suggesting that people might exploit soft robots for personal gain rather than ethical purposes, increasing societal risks.
- **Emotional Manipulation Risks**
  The human-like or animal-like appearance of soft robots was perceived as increasing the risk of emotional dependency and manipulation, potentially leading to negative personality changes or decreased social competence.
- **Increased Awareness of Risks**
  Some participants noted that reflecting on soft robots' positive attributes also highlighted potential vulnerabilities, such as the risk of misuse or unintended consequences.
- **Societal Implications**
  The perception that reliance on soft robots could reflect societal failure, with robots replacing human care in ways that undermine the importance of genuine human interaction and selflessness.
- **Concerns Over Employment and Care Dynamics**
  Participants worried that integrating soft robots into caregiving might exacerbate workforce shortages in caregiving professions, discouraging individuals from pursuing these roles.
- **Skepticism Towards New Technology**
  Some participants viewed new technology, including soft robots, with general skepticism, associating it with potential unforeseen disadvantages or societal risks.

Socially Assistive Robot, positive post CAM

- **Increased human-likeness**
  Soft robots were perceived as more human-like and emotionally engaging, often compared to a cuddly pet rather than a mechanical device, which made them feel more approachable and relatable.
- **Reduction of perceived danger**
  Participants noted that soft robots seemed less threatening and less prone to misuse compared to rigid robots, making them feel safer and more suitable for sensitive applications like therapy.
- **Expanded use cases**
  The examples provided during the exercise, such as Paro the robotic seal, helped participants envision broader, positive application scenarios like therapeutic or social interactions, enhancing their perception of soft robots.
- **Minimized negative traits**
  Participants found fewer negative aspects in soft robots compared to rigid ones. The soft design was seen as mitigating concerns related to potential harm or coldness typically associated with traditional robots.
- **Association with pets**
  The soft robots' resemblance to pets (e.g., Paro's soft, cuddly appearance and gentle movements) evoked positive emotional responses, such as comfort and curiosity, that contrasted sharply with the more mechanical image of rigid robots.
- **Shift in perspective**
  Exposure to new information and examples led participants to reevaluate their initial impressions, seeing additional benefits and removing or reducing previously noted disadvantages.

Rescue Robot, negative post CAM

- **Fragility and Susceptibility to Damage**
  Participants highlighted that soft robots are perceived as less durable and more prone to damage, especially in harsh or dangerous environments, reducing their reliability in rescue missions.
- **Technical Limitations**
  The lack of precision and technical robustness due to the use of soft materials was frequently mentioned as a disadvantage.
- **Increased Risk of Errors**
  Concerns were raised about the potential for soft robots to make incorrect decisions or face operational failures due to their design limitations.
- **Association with Negative Scenarios**
  After reflecting on the concept of soft robots, participants recalled or imagined more negative aspects and scenarios where soft robots could fail or have limited applicability.
- **Lower Versatility Compared to Rigid Robots**
  Participants believed that rigid robots are generally more advantageous and versatile in rescue situations, leading to a comparative disadvantage for soft robots.
- **Developmental Challenges**
  The need for further development and refinement of soft robots was seen as a barrier to their immediate effectiveness and usefulness.

Rescue Robot, positive post CAM

- **Reduced Risk of Harm**
  Participants perceived soft robots as posing less risk of injury to victims and patients due to their natural and gentle movements.
- **Increased Versatility**
  Soft robots were associated with more diverse use cases, including the ability to access hard-to-reach areas or provide aid in otherwise inaccessible locations.
- **Lower Potential for Misuse**
  Soft robots were viewed as less prone to misuse or ethical concerns, particularly in the absence of comprehensive ethical guidelines for AI and robotics.
- **Newly Realized Benefits**
  Exposure to additional information or examples about soft robots highlighted advantages that participants had not initially considered.

4 CODING GUIDELINES CATEGORIES

The coding guidelines categories were developed using a two-step approach combining deductive and inductive methods: (1) deductive category formation based on existing literature and (2) inductive analysis of the CAM data, whereby we identified potential new categories. These guidelines were subsequently applied to perform a qualitative content analysis of the data (R2). Graphical outputs were then generated to visualize code frequencies in ”rigid” and ”soft” CAMs.

Table S1: Coding guidelines for identifying specific risks and benefits of (soft) robots. Coding guidelines for qualitative content analysis of CAMs, focusing on specific codes that represent concepts related to a robot's operation, interaction, and impact. Each code represents a distinct concept, with a clear definition, coding rules, and examples to guide the analysis. Abbreviations are used for each code to streamline the analysis process.

Code	Definition	Coding Rules	Examples
Safety (SA)	The belief that the robot is operating safely performing tasks without posing risks and/or harm to humans performing tasks safer than humans	Compared to "Trust" this concept primarily concerned with operational safety rather than overall reliability and integrity of the robot	"Accuracy of execution"; "Safer for humans"; "Precision and efficiency"; "Reduced risk of injury"; "Quick action"
Risk (R)	The belief that the robot is operating unsafely performing tasks that pose risks to humans performing tasks riskier than humans	Compared to "Mistrust" this concept emphasizes the assessment of risky behaviors and actions on the operational level, rather than focusing on the robot's overall reliability and integrity	"Lack of accuracy"; "Slight damage"; "Possible malfunctions"
Trust (T)	The belief that the robot performs with integrity and/or reliability is thus considered trustworthy	Compared to "Safety" this concept focuses on the overall reliability and integrity of the robot, rather than focusing solely on operational safety components	"Consistency of care"; "objective/neutral"; "Adaptation to patient/client"; "better knowledge capacity"; "does not evaluate"
Mistrust (MT)	The lack of belief that the robot performs tasks with integrity and/or reliability leading to mistrust, skepticism and/or doubt	Compared to "Risk" this concept focuses on skepticism or doubts about the robot's reliability and integrity, rather than focusing on specific risky behaviors or actions on the operational level	"Discrimination against individuals"; "Favoritism"; "can be abused"; "Algorithm bias"

Continued on next page

Table S1 (continued): Coding guidelines for identifying specific risks and benefits of (soft) robots.

Code	Definition	Coding Rules	Examples
Anthropomorphism, pos. (AP)	Positive evaluation of the attribution of human characteristics and/or behaviors to the robot such as autonomy or emotions	Compared to "HRI, pos.," this concept focuses more on the evaluation of specific positive robot characteristics than on the interaction itself	"Autonomous decisions"; "Comforts the injured"; "Conversation partner for the lonely"
Anthropomorphism, neg. (AN)	Negative evaluation of the attribution or lack of human characteristics and/or behaviors to the robot such as autonomy or emotions	Compared to "HRI, neg.," this concept focuses more on the evaluation of specific negative robot characteristics than on the interaction itself	"Autonomous decisions"; "limited emotional intelligence"; "lack of empathy"
Human-Robot-Interaction, pos. (HRIP)	Positive implications of the interaction between humans and robots	Compared to "Social impact, pos.," this concept emphasizes positive aspects of the direct interaction of humans and robots, not broader societal impacts	"Interaction with elderly/autistic individuals"; "Assistance with feelings of shame (objective/neutral)"
Human-Robot-Interaction, neg. (HRIN)	Negative implications of the interaction between humans and robots	Compared to "Social impact, neg.," this concept emphasizes negative aspects of interaction, not broader societal impacts	"Unwanted emotional relationship"; "Impersonal"; "Exclusion"; "Dependency"
Social impact, pos. (SIP)	Positive impacts of robots human-robot-interaction on society	Compared to "HRI, pos.," this concept is not about the direct interaction between humans and robots but emphasizes positive impacts that robots and HRI might have on society, including long term impacts	"more job opportunities"; "promotes social interaction/integration"; "justice (objectivity)"; "reduces loneliness"

Continued on next page

Table S1 (continued): Coding guidelines for identifying specific risks and benefits of (soft) robots.

Code	Definition	Coding Rules	Examples
Social impact, neg. (SIN)	Negative impacts of robots human-robot-interaction on society	Compared to "HRI, neg.," this concept emphasizes negative impacts that robots and HRI might have on society, including long term impacts	"fewer jobs"; "loss of human nature"; "loss of human interaction"; "loss of human skills"; "coarsening of human behavior"; "people being replaced"
Technological limitation (TL)	Technological limitations of the robot	Compared to "Mistrust", "HRI, neg." & "Social impact, neg." this concept focuses solely on the technological limitations of the robot. It can be rated positively or negatively	"Limited know-how"; "limited autonomy"; "Defects"; "Limited learning ability"
Technological possibilities (TP)	Technological possibilities of the robot	Compared to "Trust", "HRI, pos." & "Social impact, pos." this concept focuses solely on the technological possibilities of the robot	"permanent readiness for deployment"; "able to overcome obstacles"
Low cost (LC)	Low cost of robot fabrication and deployment on a personal and/or societal level	Compared to "High Cost," this concept focuses on economical aspects and cost-effectiveness in robot development and deployment	"inexpensive"; "cost-efficient"; "cheap labor"
High cost (HC)	High cost of robot fabrication and deployment on a personal and/or societal level	Compared to "Low Cost," this concept highlights the higher financial investment required for robot development and deployment	"expensive"; "high costs"

5 CODING GUIDELINES CODES (WITHIN CATEGORIES)

The coding guidelines were developed through an inductive process, utilizing Large Language Models to identify overarching super-categories for individual codes. This was followed by manual refinement to enhance precision and clarity. The refined guidelines were subsequently employed, whereby single-word instances that lacked sufficient contextual information were excluded from the coding process. Graphical representations were generated to illustrate code frequencies across ”rigid” and ”soft” CAMs. These visualizations were interpreted based on the descriptive definitions of the codes and the text segments assigned to each category, providing insights into the argument structures within the single categories.

Note: Abb. indicates the abbreviations used for coding categories.

5.1 Socially Assistive Soft Robot

Table S2: Coding guidelines for identifying specific risks and benefits of (soft) robots within categories for socially assistive robots developed through an inductive process, utilizing Large Language Models. The complete list of identified codes can be found on GitHub.

Abb.	Category	Code	Description	soft	rigid
HRIN	perceived negative Human-Robot-Interaction	Dependence	Identifies instances where participants discuss reliance on socially assistive robots for various tasks, emphasizing the nuanced perspectives on the benefits and risks of dependence, including reduced human contact and emotional impacts.	19	4
AN	perceived negative anthropomorphism	Lack of Empathy	Captures the inability of socially assistive robots to convey human warmth or genuine emotional connection, highlighting a key concern in the context of perceived negative anthropomorphism.	18	3
TP	perceived technological possibilities	Availability	Captures references to the accessibility and presence of socially assistive robots beyond standard operating hours in the context of discussing the benefits and risks of their technological capabilities.	17	0
HRIP	perceived positive Human-Robot-Interaction	Target Demographic	Identifies references to specific groups or populations that socially assistive robots are designed to benefit, such as socially isolated individuals or the elderly, within the context of discussing the key benefits and risks of Human-Robot-Interaction.	16	2
HRIP	perceived positive Human-Robot-Interaction	Robotic Assistance	Identifies instances where participants highlight the positive impact of socially assistive robots in assuming degrading tasks, enhancing efficiency and quality of life.	15	0
HRIP	perceived positive Human-Robot-Interaction	Emotional Support	Identifies instances where participants express a desire for emotional support from socially assistive robots, including the emotional and sensory benefits of soft, life-like materials in facilitating interactions.	14	5

Continued on next page

Table S2 (continued): Coding guidelines within categories for socially assistive robots.

Abb.	Category	Code	Description	soft	rigid
AN	perceived negative anthropomorphism	Limitations	Highlights the acknowledgment of the limitations of robots in understanding and responding to human emotions and needs, particularly in providing genuine emotional support and connection.	13	4
HRIP	perceived positive Human-Robot-Interaction	Promote Interaction	Identifies instances where socially assistive robots are seen as encouraging social interaction, particularly for introverted or lonely individuals, fostering increased engagement with others.	13	5
HRIP	perceived positive Human-Robot-Interaction	Companion	Captures instances where participants express the potential for emotional attachment and companionship with socially assistive robots, highlighting the perceived benefits of forming a close relationship with these machines.	13	3
SA	perceived safety	Material	Highlights instances where participants evaluate the differences in materials used in socially assistive robots, with a focus on the low risk of injury during interactions or accidents due to the soft materials, leading to higher acceptance.	13	10
TL	perceived technological limitations	Limited Assistance	Denotes instances where participants express concerns about socially assistive robots being able to offer only restricted support, particularly in urgent or critical scenarios.	13	0
AP	perceived positive anthropomorphism	Material	Identifies instances where participants express a preference for socially assistive robots designed with soft, relatable materials, such as silicone, which foster physical affection and make the robot more approachable and human-like.	11	5
HRIN	perceived negative Human-Robot-Interaction	Lack of Humanity	Captures instances where participants emphasize the irreplaceable value of human empathy and connection, which cannot be adequately replicated by interactions with robots.	11	1

Continued on next page

Table S2 (continued): Coding guidelines within categories for socially assistive robots.

Abb.	Category	Code	Description	soft	rigid
SA	perceived safety	Efficiency	Identifies instances in the data where participants highlight the effectiveness and productivity of socially assistive robots in performing tasks efficiently, with arguments that robots are working faster.	11	4
TL	perceived technological limitations	Lower Adaptability	Identifies instances where participants express concerns about the restricted ability to personalize or modify socially assistive robots to suit individual needs or preferences, and their limited flexibility in adapting to different environments or changing user requirements.	10	1
AP	perceived positive anthropomorphism	Emotional Support	Captures instances where participants attribute emotional support qualities to socially assistive robots, viewing them as friends or listeners that aid in coping with thoughts and feelings, especially in challenging emotional situations.	9	6
HRIN	perceived negative Human-Robot-Interaction	Unhealthy Relationships	Identifies instances where participants express concerns about forming unhealthy dynamics with socially assistive robots, potentially leading to decreased human contact and distorted emotional interactions.	9	2
R	perceived risks	Technical Issues	Identifies instances where participants express concerns or frustrations related to malfunctions, glitches, or limitations in the technical performance of socially assistive robots, highlighting potential disruptions or challenges in their use.	9	4
TP	perceived technological possibilities	Usability	Captures references to concerns about the functionality and practicality of socially assistive robots, including issues related to technical malfunctions and potential user health impacts.	9	3
HRIN	perceived negative Human-Robot-Interaction	Superficial Interaction	Captures concerns that interactions with robots may feel insincere or lack depth compared to human connections, raising questions about the authenticity of such engagements.	8	1

5.2 Search and Rescue Soft Robot

Table S3: Coding guidelines for identifying specific risks and benefits of (soft) robots within categories for rescue robots developed through an inductive process, utilizing Large Language Models. The complete list of identified codes can be found on GitHub.

Abb.	Category	Code	Description	soft	rigid
SA	perceived safety	Safety	Emphasizes the role of rescue robots in reducing risks and protecting humans from harm, including minimizing human errors during rescue operations and enhancing safety through hazard-detecting sensors.	67	8
TP	perceived technological possibilities	Basic Needs	Identifies instances where the discussion centers around providing essential supplies like food, water, and medicine to victims, emphasizing robots' roles in ensuring survival until full rescue.	34	23
SA	perceived safety	Risk Reduction	Captures the role of rescue robots in minimizing risks for both victims and rescuers, reducing exposure to dangerous conditions.	31	9
TL	perceived technological limitations	Perceived Risk	Captures instances where participants express skepticism about the ability of AI, such as rescue robots, to accurately assess complex situations compared to human judgment.	23	5
SA	perceived safety	Efficiency	Defines the ability of rescue robots to improve operational performance by enhancing response speed and effectiveness in emergencies.	19	2
TP	perceived technological possibilities	Accessibility	Identifies instances where participants highlight the advantage of robots, particularly drones, in accessing and navigating locations that are challenging for humans to reach, including unstable areas.	18	4
R	perceived risks	Durability	Identifies instances where participants express concerns about the fragility or susceptibility to damage of rescue robots due to their soft construction, as well as material and production defects.	17	15

Continued on next page

Table S3 (continued): Coding guidelines within categories for rescue robots.

Abb.	Category	Code	Description	soft	rigid
SA	perceived safety	Speed	Focuses on the importance of rapid response and swift operations in rescue scenarios, highlighting the ability of robots to enhance efficiency and productivity in hazardous situations.	17	1
TL	perceived technological limitations	Damage not Excludable	Identifies instances where participants express concerns that damage to rescue robots may not be easily repairable or preventable. Participants highlighted that the soft material can damage quickly, raising concerns about the durability, repairability, and longevity of rescue robots in critical situations.	17	14
TP	perceived technological possibilities	Specialized Tasks	Identifies instances where rescue robots are highlighted for their unique capabilities to perform specialized tasks beyond human capacity, such as flying, shrinking, hacking doors, and transmitting images for enhanced rescue operations.	17	6
TL	perceived technological limitations	Error Risk	Identifies instances where concerns are raised about the potential for errors or mistakes in the performance of rescue robots. Errors can stem from software, hardware, or algorithmic issues, questioning their reliability and effectiveness in comparison to human responders.	16	0
SA	perceived safety	Access	Describes the capability of rescue robots to access remote or hard-to-reach locations quickly, enabling faster response times and potentially saving lives.	15	3
R	perceived risks	Perceived Risk	Identifies concerns expressed by participants regarding the potential negative outcomes associated with rescue robots, such as lack of sensitivity leading to exacerbation of situations like collapses.	13	1

Continued on next page

Table S3 (continued): Coding guidelines within categories for rescue robots.

Abb.	Category	Code	Description	soft	rigid
SA	perceived safety	Operational Capability	Describes the ability of rescue robots to function effectively in hazardous environments, demonstrating adaptability and unrestricted functionality.	13	0
SA	perceived safety	Physical Capabilities	Focuses on the ability of rescue robots to perform physical tasks beyond human capabilities, especially in environments where human performance is limited.	13	1
SA	perceived safety	Accessibility	Highlights the advantage of rescue robots in accessing tight or narrow spaces, showcasing their adaptability and potential to enhance safety.	13	3
TL	perceived technological limitations	Specialization	Identifies instances where participants highlight the limited scope of rescue robots, emphasizing their specialization for particular rescue tasks while noting their limited adaptability to diverse rescue scenarios.	13	3
TL	perceived technological limitations	Faulty Analyses	Identifies instances where participants express concerns about potential errors or inaccuracies in the analysis performed by rescue robots due to reliance on recognition software. Highlights the need for human oversight to prevent errors, emphasizing the importance of human judgment in critical situations.	13	1
R	perceived risks	Potential Harm to Victims	Identifies concerns related to the possibility of harm to individuals caused by technical malfunctions in rescue robots, such as potential risks of injury or even fatality.	12	5
SA	perceived safety	Strength	Describes the physical superiority of rescue robots, including their ability to lift heavy objects and demonstrate strength beyond human capacity, particularly in challenging environments.	12	0
